# Early Utilization of Intravenous Immunoglobulin in Heparin-Induced Thrombocytopenia for Limb Salvaging Purposes

**DOI:** 10.7759/cureus.23202

**Published:** 2022-03-15

**Authors:** Sarah Abu Kar, Amandeep Kaur, Ahmed M Khan, Dennis Bloomfield

**Affiliations:** 1 Internal Medicine, Richmond University Medical Center, Staten Island, USA; 2 Hematology and Oncology, Richmond University Medical Center, Staten Island, USA; 3 Radiology, Richmond University Medical Center, Staten Island, USA; 4 Research, Richmond University Medical Center, Staten Island, USA

**Keywords:** rapid-onset hit, platelet factor 4, hypercoagulable state, venous thromboembolism, argatroban, serotonin release assay, ivig, autoimmune hit

## Abstract

We present a case of a 28-year-old diabetic female who presented with high-burden lower extremity deep vein thrombosis (DVT) after previous exposure to unfractionated heparin (UFH). Heparin was discontinued, and non-heparin parenteral anticoagulant, argatroban, was started based on a high clinical suspicion of heparin-induced thrombocytopenia with thrombosis (HITT). The diagnosis of HIT was later proven by positive immune and functional assays. The severity of thrombocytopenia and the need for surgical intervention to salvage the limb prompted the use of intravenous immunoglobulin (IVIG) early on in the treatment course to recover platelet counts, halt the prothrombotic state, and prepare the patient for thrombectomy. The patient was put on direct oral anticoagulants (DOACs), apixaban, after thrombectomy, and platelet count recovery with no new thrombosis or bleeding episodes was reported after three months of follow-up.

## Introduction

Heparin-induced thrombocytopenia (HIT) is a rare complication due to heparin exposure where the body generates antibodies against platelet factor 4 (PF4) and heparin that activates platelets and generates a prothrombotic state [1]. Many case reports in the literature described the use of intravenous immunoglobulin (IVIG) to halt the thrombotic state after failure of non-heparin parenteral anticoagulation mostly in cases of delayed-onset and persistent HIT. Very few cases described its early use in the course of the disease. We present a case of HIT that caused a significant thrombotic burden in the lower extremity requiring early intervention (thrombectomy) and the early use of IVIG to recover platelet counts overcoming parenteral anticoagulation.

## Case presentation

The patient is a 28-year-old female with a past medical history of diabetes mellitus who presented to the hospital with right lower extremity pain, numbness, and swelling. Two weeks preceding the admission, the patient was admitted for management of hypertriglyceridemia-induced pancreatitis for which she needed apheresis through a central venous catheter in the right femoral vein and received deep vein thrombosis (DVT) prophylactic treatment with unfractionated heparin (UFH) 5,000 units subcutaneously every 12 hours. On this admission, her vital signs were within normal limits. Her physical examination was only pertinent for right lower extremity swelling; the circumferential girth of the above and below the knee measurements was three times that of the left leg.

**Figure 1:**
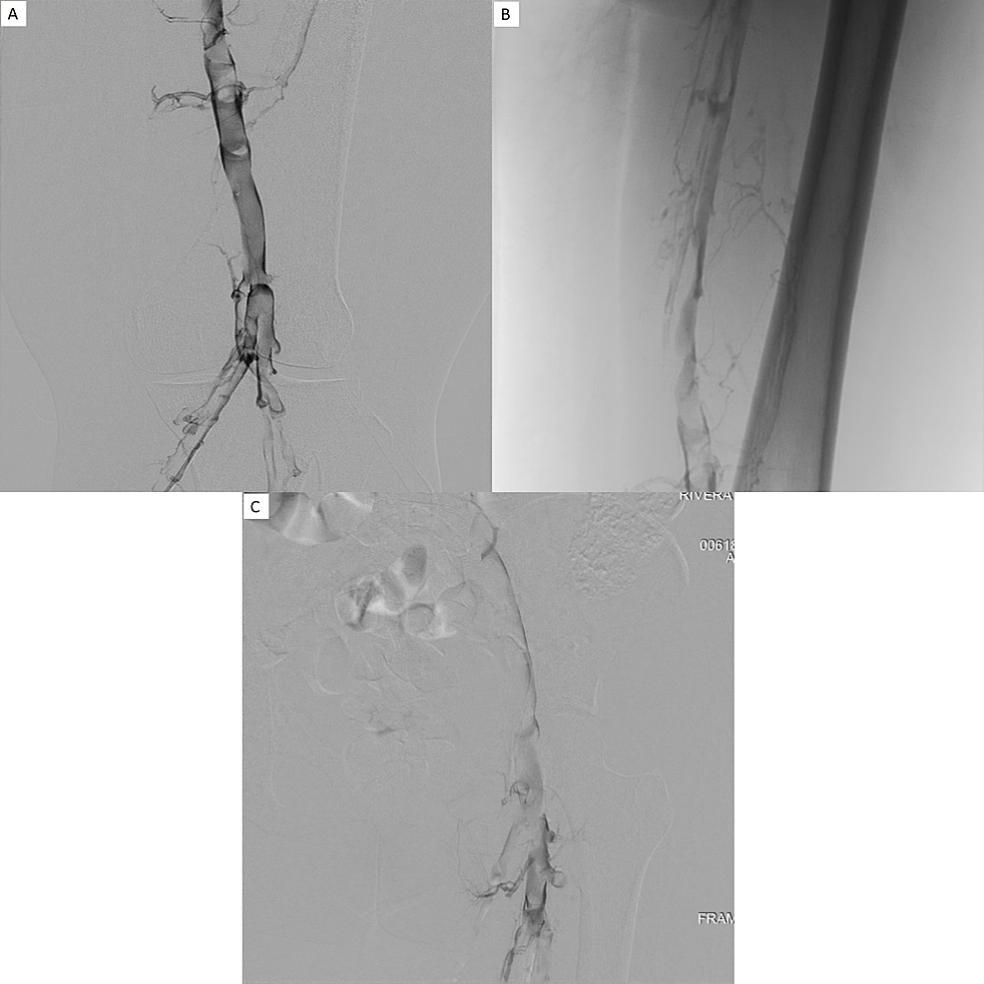
A, B, and C: Right lower extremity venogram shows extensive clot involving the right popliteal, femoral, and iliac veins with extensive collateralization.

The venous duplex study of lower extremity showed acute DVT in external iliac, common femoral, superficial femoral, popliteal, posterior tibial, peroneal, and greater saphenous vein (Figure 1A-1C). She was started on heparin continuous infusion. Her platelet counts dropped from 98,000 to 34,000 on day 3 of the admission (Table 1).

**Table 1:** Platelet counts of the patient during admission. IVIG was given on days 3 and 4.

Days	Day 1	Day 2	Day 3	Day 4	Day 5	Day 6	Day 7	Day 8	Day 9	Day 10
Platelet count	98,000	63,000	34,000	28,000	35,000	60,000	105,000	93,000	112,000	118,000

**Figure 2:**
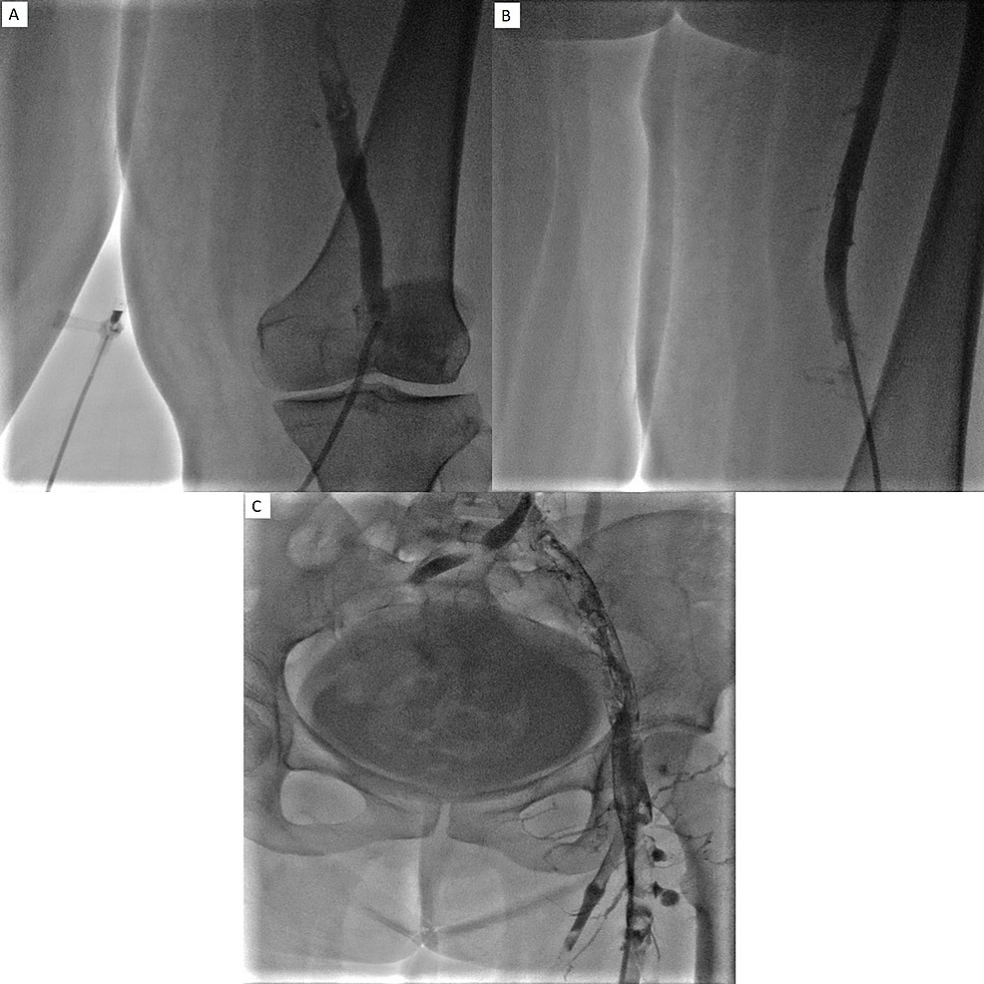
A, B, and C: S/p right lower extremity catheter-guided thrombolysis and suction thrombectomy. Moderate improvement in lower extremity clot burden. However, a residual clot was present, especially within proximal deep venous structures, so it was decided to keep the catheter in place and administer 1 mg tPA per hour.

**Figure 3:**
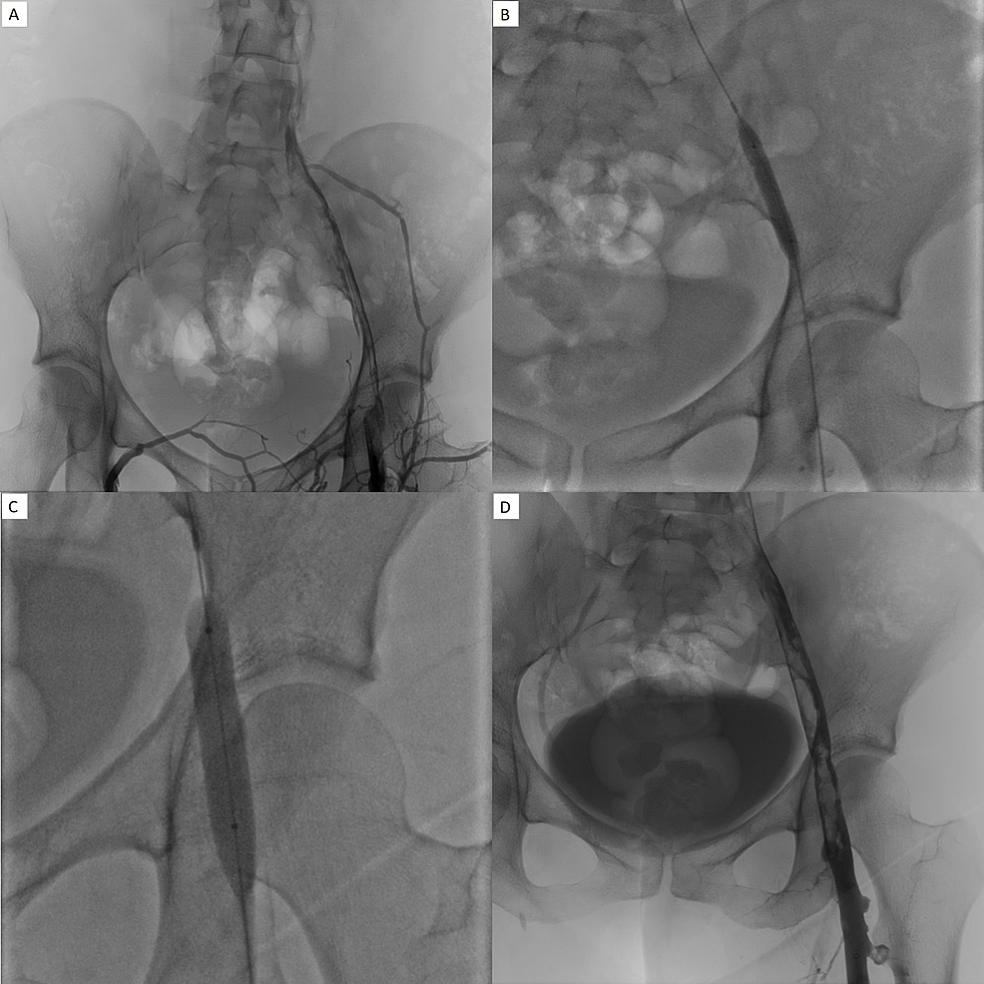
A, B, C, and D: The patient returned 17 hours later as there was bleeding at the catheter site. tPA administration was stopped promptly, and the patient returned to IR for a right lower extremity venogram and further management. Improved clot burden from prior; however, there was still moderate residual clot within the proximal IVC, iliac vein extending into the mid-femoral vein (A). Angioplasty with 8 × 4 mm and 10 × 4 mm balloons was performed (B and C); however, due to residual thrombus, mechanical suction thrombectomy was performed. Post-procedural images show further improved clot burden with some minimal residual clot, and it was decided to leave the catheter in place, administering 0.5 mg tPA per hour (D).

**Figure 4:**
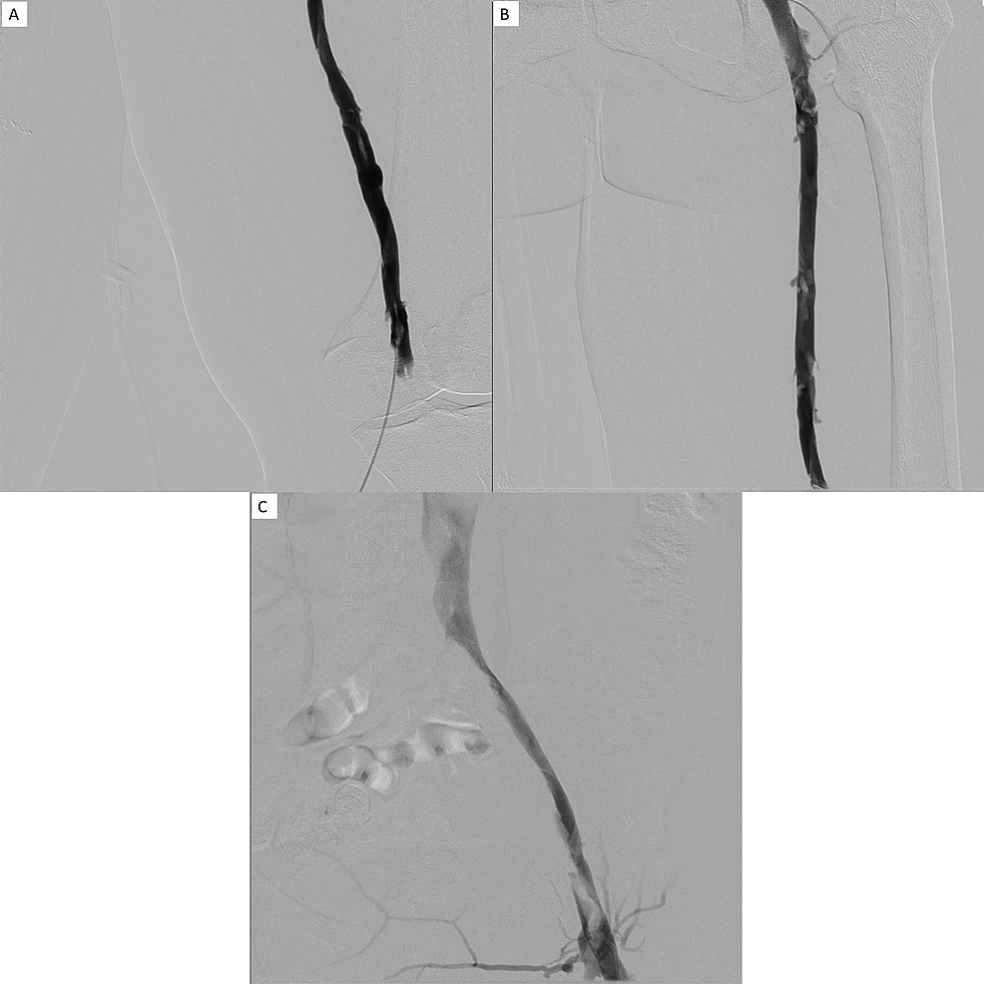
A, B, and C: Follow-up 26 hours after initial intervention showed continued improvement in clot burden. There was however residual thrombus and stenosis within the iliac and femoral veins, and repeat angioplasty with 10 French balloon was performed. Suction thrombectomy was again performed as previously described. Final venogram showed significant improvement in overall clot burden and luminal caliber with minimal nonocclusive thrombus seen in the iliac venous system.

The likelihood of HIT was very high; thus, heparin was discontinued, and the patient was started on argatroban continuous infusion. HIT workup included heparin-induced platelet antibodies, which were strongly positive, and serotonin release assay, which showed 89% and 90% release at 0.1 UI/mL and 0.5 IU/L of unfractionated heparin (UFH) and 0% release at 100 IU/mL of UFH, respectively. On day 3 of argatroban infusion, the patient had minimal improvement in her platelet count. Her symptoms persisted and included lower extremity pain and swelling. Mechanical thrombectomy was undertaken (Figures 2-4) for limb saving purposes due to the high burden of thrombosis in the right leg. A decision was taken to give IVIG 0.5 g/kg twice over two days to help raise the platelet count, which improved from 28,000 to 105,000 within three days, and the patient underwent catheter-directed tPA thrombolysis and thrombectomy with no intraoperative and postoperative complications (Figures 2-4). Her symptoms improved, and her platelet count normalized to >150,000 by day 11 since the onset of HIT. She was subsequently discharged on apixaban (Eliquis) 5 mg PO twice daily with home physical therapy. The patient followed up at the outpatient clinic for six months at two months intervals with good compliance and tolerance to oral anticoagulation and no evidence of bleeding or new thrombosis.

## Discussion

Our patient developed HIT after exposure to unfractionated heparin, causing a huge venous thrombosis burden in the lower extremity that imposed a high risk of limb gangrene. The decision to use IVIG was solely to halt the progression of thrombi in the leg and facilitate early surgical intervention.

Very few case reports have described the use of IVIG in the treatment of HIT. A little more than 30 cases were reported in the literature. It has been proposed that IVIG can be used for the treatment of HIT in specific situations where rapid inhibition of HIT antibody activity is required, such as cases of sinus vein thrombosis, critical limb ischemia, and severe or persistent thrombocytopenia [2]. IVIG were also used in cases of delayed onset HIT, persistent HIT, fondaparinux HIT, flush HIT, and spontaneous HIT [3]. The fact that IVIG treatment carries thrombotic risk on its own argued against its use initially for HIT treatment [4,5], but subsequently, in vitro studies proved the inhibiting ability of IVIG to platelet-activating HIT antibodies [6-8], which facilitates its reemergence as a treatment option.

Dosages were given at 0.5 g/kg to 2 g/kg over two to five days. Most of the responses were good to excellent. Only three cases reported had no response to treatment, and one case had subsequent thrombosis. However, it was argued that the argatroban dosage was not optimized [9-11].

No randomized controlled studies are available to compare the effects of the treatment of HIT with direct oral anticoagulants (DOACs) versus warfarin on thrombosis and thrombosis-related mortality, and bleeding and bleeding mortality [12]. Warkentin reviewed 46 patients with confirmed or acute HIT in whom DOACs (rivaroxaban, dabigatran, edoxaban, and apixaban) were used [1]. Only one patient had thrombosis while on treatment with rivaroxaban, and no bleeding events were reported. Sixteen other cases were reviewed where DOACs were used either initially or as a transition from non-heparin parenteral anticoagulation, and one patient developed thrombosis while on rivaroxaban [12]. Of note, patients who receive DOACs for HIT treatment generally have a better prognosis.

Our patient was discharged on Eliquis since she already received thrombectomy and to avoid the need for frequent visits to Coumadin Clinic. The patient was still asymptomatic after three months of treatment on apixaban with no evidence of thrombosis or bleeding.

## Conclusions

This is a case of HIT complicated with a high thrombus burden in the limb that may have had jeopardized its viability where the utility of IVIG proved to be limb-saving. It was used early on in the treatment along with argatroban infusion. The patient tolerated the treatment well with no evidence of thrombus expansion. Despite the scarcity of evidence, IVIG treatment is repeatedly proving to be beneficial in all case reports cited in the literature and should be considered in special situations. On top of that, our patient tolerated DOAC well post-IVIG and argatroban infusion and did not manifest any thrombus progression or bleeding.
